# A Novel LINS1 Truncating Mutation in Autosomal Recessive Nonsyndromic Intellectual Disability

**DOI:** 10.3389/fpsyt.2020.00354

**Published:** 2020-05-18

**Authors:** Babylakshmi Muthusamy, Anikha Bellad, Pramada Prasad, Aravind K. Bandari, G. Bhuvanalakshmi, R. M. Kiragasur, Satish Chandra Girimaji, Akhilesh Pandey

**Affiliations:** ^1^Institute of Bioinformatics, Bangalore, India; ^2^Center for Molecular Medicine, National Institute of Mental Health and Neurosciences (NIMHANS), Bangalore, India; ^3^Manipal Academy of Higher Education, Manipal, India; ^4^Department of Neurology, National Institute of Mental Health and Neurosciences (NIMHANS), Bangalore, India; ^5^Department of Child and Adolescent Psychiatry, National Institute of Mental Health and Neurosciences (NIMHANS), Bangalore, India; ^6^Department of Laboratory Medicine and Pathology, Mayo Clinic, Rochester, MN, United States; ^7^Center for Individualized Medicine, Mayo Clinic, Rochester, MN, United States

**Keywords:** autosomal recessive, truncating mutation, WNT signalling, Embryogenesis, genetic disorders

## Abstract

The large majority of cases with intellectual disability are syndromic (i.e. occur with other well-defined clinical phenotypes) and have been studied extensively. Autosomal recessive nonsyndromic intellectual disability is a group of genetically heterogeneous disorders for which a number of potentially causative genes have been identified although the molecular basis of most of them remains unexplored. Here, we report the clinical characteristics and genetic findings of a family with two male siblings affected with autosomal recessive nonsyndromic intellectual disability. Whole exome sequencing was carried out on two affected male siblings and unaffected parents. A potentially pathogenic variant identified in this study was confirmed by Sanger sequencing to be inherited in an autosomal recessive fashion. We identified a novel nonsense mutation (p.Gln368Ter) in the *LINS1* gene which leads to loss of 389 amino acids in the C-terminus of the encoded protein. The truncation mutation causes a complete loss of LINES_C domain along with loss of three known phosphorylation sites and a known ubiquitylation site in addition to other evolutionarily conserved regions of LINS1. *LINS1* has been reported to cause MRT27 (mental retardation, autosomal recessive 27), a rare autosomal recessive nonsyndromic intellectual disability, with limited characterization of the phenotype. Identification of a potentially pathogenic truncating mutation in LINS1 in two profoundly intellectually impaired patients also confirms its role in cognition.

## Introduction

Intellectual disability (ID) is a neurodevelopmental disorder characterized by significant limitations in intellectual functioning and adaptive behavior, which include conceptual, social, and practical skills. A meta-analysis of ID estimated its prevalence as ~10 in 1,000 population with differences based on age and income (1). A higher proportion of males, children/adolescents and people from low-middle income countries are reported to be affected with ID (1). ID is classified into syndromic ID and nonsyndromic ID based on the clinical presentation of patients. In syndromic ID, patients exhibit one or more distinct clinical features or comorbidities in addition to ID. Nonsyndromic ID is generally a pure form of ID which is defined by the presence of ID as the sole clinical feature. However, the distinction between syndromic and nonsyndromic ID is often blurred owing to overlap and subclinical presentation (2). More than 200 candidate genes have been associated with nonsyndromic ID thus far (3).

Next generation sequencing has accelerated our understanding of genetic variants that underlie unexplained inherited genetic disorders. Identification of mutations through whole exome sequencing has enabled molecular diagnosis of genetic disorders. However, there are still a number of neurodevelopmental disorders whose molecular basis remains unknown because of the rare nature of such diseases. Autosomal recessive ID (ARID) is one such group of disorders which is poorly elucidated because of a high degree of clinical and genetic heterogeneity (4). As with other autosomal recessive disorders, ARID is frequent in some regions of the world where consanguineous marriages are common (e.g. some parts of India) (4). Genes causing ARID have been estimated to be run into thousands and most of them remain unknown (4). More than 40 candidate genes are known to be associated with nonsyndromic ARID and a large majority of them were identified through next generation sequencing methods (4). Currently, there are 61 entries that correspond to nonsyndromic ARID reported in OMIM (https://www.omim.org/graph/linear/PS249500) which were identified through linkage analysis and homozygosity mapping. The exact gene responsible for almost a quarter of these still remain unknown despite advancements in finding genetic causes of these disorders using next generation sequencing.

In this study, we describe an Indian family with two affected siblings with profound ID. The genetic analysis using next generation sequencing led to identification of a novel autosomal recessive nonsense mutation in *LINS1* gene which may play crucial role in cognition. *LINS1* has previously been identified as a genetic cause for MRT27 (mental retardation, autosomal recessive 27) which has been reported in a small number of patients with limited characterization of their phenotypic features. Here, we report a novel truncating mutation in LINS1 along with detailed clinical characteristics of the affected patients.

## Materials and Methods

### Ethics Statement

Two male siblings affected with profound ID and their parents were recruited for this study. Diagnosis was carried out by experienced clinicians based on ICD-10 criteria. This study was approved by the Ethics Committee at the National Institute of Mental Health and Neurosciences (NIMHANS), Bangalore. Written informed consent to participate in this study was provided by the parents of the patients.

### Cytogenetics and Exome Sequencing

DNA was extracted from peripheral blood using QIAamp DNA minikit (Qiagen) following the manufacturer's protocol. Karyotyping was carried out on blood samples of patients using standard procedures to look for chromosomal abnormalities. Whole exome sequencing of the patients and parents were carried out to look for single nucleotide variants and small indels. Karyotyping, sequencing library preparation, exome capture, whole exome sequencing and data analyses were carried out as described previously (5). Briefly, whole exome capturing and enrichment was carried out using SureSelectXT Human All Exon V5+UTR. The captured library DNA was sequenced on Illumina HiSeq X10 platform (Illumina, USA) to generate paired end (2×150 bases) DNA sequencing reads. Quality of the raw sequencing data was checked using FastQC toolkit (https://www.bioinformatics.babraham.ac.uk/projects/fastqc/). After performing appropriate quality check followed by adapter removal and low base quality trimming, the raw reads were aligned to human reference genome (GRCh37/hg19) using Burrows-Wheeler Aligner (BWA-mem) (6). PCR duplicates were marked using Picard tools (https://broadinstitute.github.io/picard/) and post alignment quality control was performed using the genome analysis toolkit (GATK) applying GATK's best practices workflow for DNA sequencing data (7). Variant calling was carried out using GATK HaplotypeCaller and GVCF files were generated. Further, joint variant calling was performed across patients and parents samples using GATK to enhance the number of identification of mutations across the datasets. The identified variants were annotated using ANNOVAR (8) applying gene-based and filter-based annotations. Under filter-based annotations, minor allele frequency from 1,000 genomes project (9), Exome Aggregation Consortium (ExAC) (10) and gnomAD (https://www.biorxiv.org/content/10.1101/531210v2) were annotated for all identified variants (8).

### Filtering of Variants

Rare variants with minor allele frequencies of <0.01 were retained after comparing with 1,000 genomes project, ExAC, and gnomAD variants. SIFT (11), Polyphen-2 (12), MutationTaster-2 (13) and CADD (14) software tools were used to predict the functional impact of each mutation on the structure and function of the protein product. Further filtering was carried out by applying a pattern of autosomal recessive inheritance and X-linked recessive inheritance patterns in the variants identified from the patients and parents. The potential ability of the identified variants to cause the phenotype was analyzed manually using several additional bioinformatics tools and literature curation.

#### Copy Number Variants

We looked at copy number variations (CNVs) in the whole exome sequencing data using ExomeDepth tool (version 1.1.15), a freely available and widely used R package (15). ExomeDepth requires reference BAM files to combine and build a reference data set that is required to optimize and maximize the accuracy of detecting the CNVs. To build this reference dataset, we used BAM files of twelve unrelated samples that were generated using similar laboratory and computational protocols. These samples were obtained from patients of Parkinson's disease with Indian origin (unpublished in-house data). We used exonic regions of human (hg19) extracted from Ensembl database version 71 as target regions for detecting the CNVs. Default parameters of ExomeDepth were used to first generate the read counts for each exon for each sample and the same were used for the detecting CNVs. We identified CNVs for autosomal chromosomes and X-chromosome separately. This was repeated for each of the four samples separately. The CNVs that were found in both the affected siblings were checked for the pathogenic or polymorphic nature by comparing them with the publicly available databases DECIPHER (16), the database of genomics variants (DGV) (17) and dbVar (18). Based on read depth and allele frequencies copy numbers were estimated and segregation analysis was carried out across all four samples using autosomal dominant, autosomal recessive or X-linked recessive inheritance patterns.

### Targeted Sanger Sequencing

In order to validate the autosomal recessive *LINS1* mutation identified through whole exome sequencing, we carried out Sanger sequencing of DNA obtained from both affected siblings and obligate carrier parents. We first designed primers for a target region of 576 bp that includes the mutation site of *LINS1* gene in chromosome 15 using Primer Quest Tool (https://eu.idtdna.com/Primerquest/Home/Index). Further, the target region was amplified by performing PCR using a forward primer: 5'-CATCGCCTCCAGAATCCA-3' and a reverse primer: 5'- GTTACAAATAGGATAACATACAGC -3'. We have used Q5 high fidelity buffer and polymerase for amplifying the target region and annealing temperature of 59°C was used. The PCR products were cleaned using a Qiaquick PCR purification kit and the product was subjected to Sanger sequencing. The chromatograms were analyzed manually to visualize the *LINS1* mutation to study the segregation of the mutation in the patients and unaffected father and mother.

## Results

### Clinical Presentation

**Figure 1:**
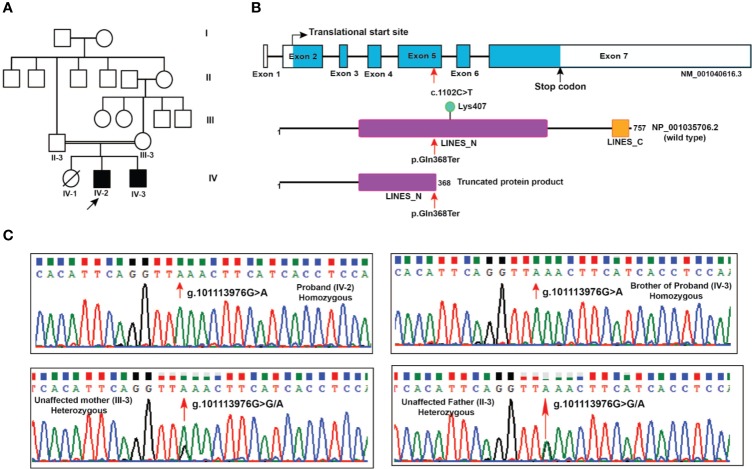
Pedigree and *LINS1* mutation. **(A)** Pedigree depicting affected and unaffected members of the proband's family. Whole exome sequencing was carried out in two affected siblings (IV-2 and IV-3) and unaffected parents (II-3 and III-3). **(B)** Schematic representation of LINS1 truncating mutation p.Gln358Ter at the genomic, cDNA, and protein levels. The location of the single nucleotide variant c.1102C > T at exon 5 in *LINS1* gene that led to a premature stop of the protein product p.Gln368Ter is indicated by arrows (red). The wild type (1-757) and mutant protein product (1-367) of LINS1 is depicted. The truncated protein product shows the partial loss of LINES_N domain and complete loss of LINES_C domain and a ubiquitylation site Lys407. **(C)** Chromatograms depicting the Sanger sequencing results of a region spanning *LINS1* mutation (g. 101113976G > A) in both the siblings (homozygous), unaffected mother (heterozygous), and unaffected father (heterozygous). The location at which mutation occurs is depicted by the arrow (red).

Two male siblings with profound ID born in a consanguineous family from the state of Karnataka, India were evaluated (**Figure 1A**). Their father (II-3) was third born of six children (five males and one female) and married his sister's daughter (second degree consanguineous parentage). Patient 1 (IV-2) is elder of the two siblings, currently aged 20 years and was first evaluated clinically at ~12 years of age. Prenatally, the mother was treated for pregnancy-induced hypertension. He was born pre-term at 34 weeks through assisted vaginal breech delivery. His birth weight was 1.8 kg. There was a delayed birth cry and the baby was kept in the incubator for one day. Developmentally, all milestones were delayed: independent walking was at 2 years, speaking a few words at 10 years, indicating toilet needs at 8 years with very poor imitative and social interaction skills. His social quotient on Vineland Social Maturity Scale was 9 at 12 years, indicating profound ID. On physical examination, he was noted to have coarse facial features, malformed dentition, long tapering fingers, wasting of pectoral muscles, and hyper-extensible finger joints. Behaviorally, he was noted to have autistic features such as very poor eye-to-eye contact, poor response to name call, poor social reciprocity, and motor stereotypies. Tandem mass spectrometry-based analysis of blood for metabolic abnormalities and urine for abnormal metabolites was performed and no abnormalities were found.

Patient 2 (IV-3) is younger of the two affected siblings (currently aged 18 years) who initially presented with complaints of seizures, severe hyperactivity and gross developmental delay at 8 years of age. During the prenatal period, the mother was diagnosed to have pregnancy-induced hypertension and cervical incompetence and was treated. He was born at full term in a hospital through normal vaginal delivery and his birth weight was 2 kg. He had generalized tonic/clonic seizures on the second day after birth. Until the age of 4 years, he continued to have 3-4 generalized seizures per month. He had gross delay in all domains of development, social, and speech being more severely affected than gross motor development: independent walking at 3 years, speaking two words at 5 years and eating independently at 8 years. He had profound ID with a social quotient of 16.6 and poor social reciprocity. Physical examination revealed a flat nasal bridge, upturned nose, large ears, coarse facial features, long slender fingers, hallux valgus and dragging gait. Behaviorally, he was noted having severe hyperactivity and inattention. In addition, he had several autistic features, poor eye-to-eye contact, and motor stereotypies in the form of sideward movements of the head. For seizures, he was given phenytoin and carbamazepine with good control. His hyperactivity was treated initially with clonidine with poor response and later with risperidone and lithium with moderate response. The phenotypic features of these patients were compared with the phenotypic features of previously reported patients with *LINS1* gene mutation (**Table 1**).

**Table 1:** Phenotypic features of the two affected siblings compared with a previously reported family [Akawi et al. (19)].

Features	Current study		Akawi et al. (19) subjects	
	IV-2	IV-3	1^st^ child	2^nd^ child
Age in years (current)	20	18		
Age at initial clinical evaluation	12 years	8 years	9 years	3 years
**Historical details**				
Prenatal period	Pregnancy-induced hypertension	Pregnancy-induced hypertension and cervical incompetence	Mild hypertension and gestational diabetes	Uneventful
Labor	Assisted vaginal breech delivery	Normal vaginal delivery	Induced	Normal
Low birth weight	1.8 kg	2.0 kg	3.0 kg	3.0 kg
Seizures	No	Yes, Since 2 years of age	No	
**Developmental details**				
Independent walking	2 years	3 years	19 months	20 months
Speaking 2 words	10 years	5 years	No speech	No speech
IQ/SQ	SQ=9 (12 years age)	SQ=16.6 (9 years)	No details	No details
Degree of intellectual disability	Severe	Profound	No details	No details
Autistic features	Yes	Yes	Absent	Absent
Hyperactivity	No	Yes	Yes	No details
Motor stereotypes	Yes	Yes	Yes	?Yes
**Physical findings**				
Facies	Coarse	Coarse	Flat mid face	Flat mid face
Long tapering Fingers	Yes	Yes	No details	No details
Weight	26 kgs	20 kgs	No details	No details

### A Novel Nonsense Mutation in LINS1 Gene

Chromosome analysis of cultured peripheral blood from both affected siblings revealed an apparently normal male 46, XY chromosome complement. Whole exome sequencing was carried out on the siblings and their unaffected parents. Around 50–60 million reads were generated, of which >98% of the paired-end reads were properly paired and aligned to the human reference genome (GRCh37/hg19). We obtained a mean depth of 72× across the four individuals. Joint variant calling resulted in a total of 259,949 variants in all four individuals. Exonic and splice site variants were retained and variants with minor allele frequency greater than 0.01 were discarded after comparing the allele frequency values reported in ExAC, 1,000 genomes project and genomAD and we obtained 1,373 rare variants. Various modes of inheritance were applied to further filter the variants. The consanguineous ancestry suggested an X-linked recessive or ARID (**Figure 1A**). We did not identify any significant X-linked recessive variants that could result in the phenotypes observed in the patients. Applying filtering using the autosomal recessive mode of inheritance, we identified gene mutations in *CASKIN2*, *SLC25A10*, *NARF*, *ELANE* and *LINS1* (**Table 2**). *CASKIN2* gene encodes for CASK interacting protein 2, a neuronal scaffold protein. Although CASKIN2 is named after its possible interaction with CASK, CASKIN2 does not interact with CASK due to the lack of CASK interacting domain (CID) (20). We identified a novel missense variant altering a valine residue to methionine at position 410 (p.Val410Met) which is predicted to be a benign variant by SIFT and Polyphen-2. This mutation is located in a region where CID domain was supposed to be present in CASKIN2. Because of the lack of CID domain in CASKIN2 which is required for CASK binding, CASKIN2 may not play direct role in CASK mediated scaffolding. In addition, CASK mediated scaffolding is achieved through the C-terminal SAM domains (21). Thus, the benign mutation p.Val410Met identified in CASKIN2 is likely nonpathogenic mutation. Solute carrier family 25 member 10 (SLC25A10) is a mitochondrial dicarboxylate carrier protein with no reported role in brain related functions. In mouse brain tissues, *SLC25A10* levels were found to be lower which may not contribute to the mitochondrial dysfunction in brain (22). Thus, *SLC25A10* mutation is not likely to be associated with the phenotypes observed in the patients. Another mutation p.Ser89Leu (RefSeq: NP_036468.1) has been identified in nuclear prelamin A recognition factor (NARF) which has been reported to be associated with mitochondrial dysfunction and iron accumulation in multiple sclerosis (23). There are no phenotypic correlations observed in patients with *NARF* mutations. Another mutation p.Arg143Cys has been identified in elastase, neutrophil expressed (ELANE) protein that was predicted to be deleterious by SIFT and Polyphen-2. ELANE is associated with neutropenia and there is no report on brain related functions. Overall, *CASKIN2, SLC25A10, NARF*, and *ELANE* mutations bear no correlation with the phenotypes observed in the patients. Of note, *LINS1* have been reported previously in patients with similar phenotypes observed in our patients establishing the genotype-phenotype correlation. Thus, *LINS1* is the potentially pathogenic mutation for the phenotypes observed in our patients.

**Table 2:** Autosomal recessive mutations with <0.01 minor allele frequencies in 1,000 genomes project, ExAC, and gnomAD databases.

Gene	Chromo some	Genomic mutation	mRNA change	Protein Change	Maximum of minor allele frequencies in databases
*LINS1*	Chr15	g. 101113976G > A	NM_001040616:exon5: c.C1102T	p.Gln368Ter	0
*CASKIN2*	Chr17	g. 73500739C > T	NM_020753:exon12: c.G1228A	p.Val410Met	0.0046
*SLC25A10*	Chr17	g. 79684843C > T	NM_001270953:exon9: c.C574T	p.Pro192Ser	0.0065
*NARF*	Chr17	g. 80426653C > T	NM_012336:exon4: c.C266T	p.Ser89Leu	0.0021
*ELANE*	Chr19	g. 855624C > T	NM_001972:exon4: c.C427T	p.Arg143Cys	0.0001

The nonsense mutation identified in *LINS1* is a single nucleotide variant g.101113976G > A that resulted in a cytosine to thymine change (c.1102C > T) in the cDNA of *LINS1* gene that is located on chromosome 15 at 15q26.3. This variant was not reported in gnomAD, ExAC or 1000 genomes project. This variant was found in homozygous state in affected individuals and heterozygous state in unaffected parents. The change in exon 5 of *LINS1* resulted in change of glutamine (CAA) to a stop codon (TAA) which would lead to premature termination of the protein product at position 368. *LINS1* gene has seven exons and the protein product of *LINS1* is a 757 amino acid long polypeptide comprising of two ‘lines' homology domains LINES_N (194-542) and LINES_C (712-747) (RefSeq accession: NP_001035706.2). The truncating mutation identified in this study (p.Gln368Ter) is located at the LINES_N domain resulting in a partial loss of LINES_N domain (194-367), complete loss of LINES_C domain (712-747) and a loss of a ubiquitylation site at position Lys407 (**Figure 1B**).

*LINS1* was reported to have only one transcript in RefSeq in the human genome build GRCh37 (hg19) and the GRCh38 version has reported with three alternatively spliced forms and several predicted and noncoding transcripts. Exon 5 in transcript NM_001040616.6 (reference transcript used in this study) was consistently intact in all isoforms including the predicted and noncoding transcripts at this location. Thus, the effect of this truncating mutation will be applicable to all protein-coding transcripts currently available in RefSeq. Sanger sequencing of the patients and the unaffected parents showed homozygous *LINS1* mutation in patients and heterozygous variant in unaffected father confirming the pattern identified using whole exome sequencing (**Figure 1C**).

### Variant Pathogenicity and Conservation Analysis

**Figure 2:**
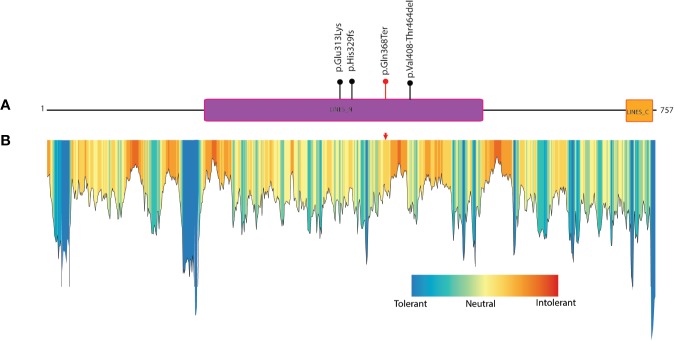
Known mutations in LINS1 gene and depiction of conservation of the truncated region of LINS1 protein. **(A)** Depiction of currently known pathogenic mutations in LINS1. The mutations reported previously are shown as black circles and the mutation identified in this study is represented by a red circle. **(B)** MetaDome analysis of full length LINS1 protein depicting all possible tolerant, intolerant and neutral amino acids. The mutation identified in this study is depicted by a red arrow in the plot. The mutation is located in a moderately intolerant location notably, several moderately and highly intolerant sites are located downstream of the mutation reported in this study at position 368.

The protein truncation mutations are potentially pathogenic mutations as they may lead to loss of several domains and functionally important regions of the protein which directly impact the protein function. The LINS1 truncation mutation identified in this study led to the deletion of 389 amino acids in the C-terminus of the protein (**Figure 1B**). The currently known mutations and the mutation identified in this study is depicted in **Figure 2A**. In order to study the functionally important regions in the deleted regions, we carried out a MetaDome analysis and protein conservation analysis across species. MetaDome (24) is a tool to predict the tolerance of the genetic mutations which in turn predicts the pathogenicity of those mutations based on the population variation data from ExAC (10) and gnomAD (https://www.biorxiv.org/content/10.1101/531210v3) as compared with the disease causing variants from the databases such as Human Gene Mutation Database (HGMD) (25) and ClinVar (26) that are mapped on the Pfam domains. MetaDome was used to visualize the potentially genetically intolerant sites/regions that could have potentially affected the protein function (**Figure 2B**).

### Copy Number Variants Analysis

We have used whole exome sequencing data of the proband, brother, father and mother as test dataset and twelve unrelated Indian samples as reference dataset for detecting CNVs. Read counts of each exons (hg19 – Ensembl version 71) were first retrieved and used for identifying the CNVs. We identified 88, 94, 83, and 94 CNVs with correlation value > 0.98 in proband, brother, mother and father's samples, respectively. Of the identified CNVs, only 32 CNVs were found in both the affected siblings and 12 were found in all four samples. Of the 12 CNVs, seven were deletions and five were duplications. These variants were found in all four samples with similar allelic frequency indicating possibly polymorphic CNVs. Thus, there is no significant CNV that is potential candidates as a cause of ID in these patients.

## Discussion

### LINS1 Mutations in MRT27

Mutations in *LINS1* gene have been previously reported to cause MRT27, a rare autosomal recessively inherited nonsyndromic ID disorder. The locus for MRT27 was first identified as 15q23–q26 in a consanguineous Syrian family using homozygosity mapping (27). In this family, five affected members including a female patient were described with the clinical characteristics of including normal motor development, delayed speech, moderate ID and epilepsy (27). *LINS1* gene as the molecular cause for MRT27 was first described by Najmabadi et al. using homozygosity mapping and next generation sequencing in a consanguineous family comprising of four affected individuals with moderate ID and microcephaly (28). Subsequently, a consanguineous family with nonsyndromic intellectually disabled patients was identified with a homozygous truncating mutation p.His329fs that terminates the protein product at position 329 (28). These two families were reported part of large cohort of patients with ARID with consanguinity and the patients were described with inadequate clinical descriptions. An Indian family has been reported with a missense mutation in LINS1 in two siblings with nonsyndromic ID, mutism and cognitive developmental delay (29). More recently, an Emirati family comprising of a male child and a female child affected with ID has been described with detailed clinical phenotypic information, of which several of them overlap with the patients described in this study (19). A homozygous deletion of five nucleotides involving a splice site was identified in this study resulting in skipping of exon 5 and splicing of exon 4 with exon 6. The loss of exon5 results in deletion of a region of 211-407 in LINS1 protein and found to be deleterious (19). The overlapping phenotypic features observed were: (i) pregnancy complications due to hypertension and induced delivery, (ii) neonatal death in the family of unknown cause, (iii) poor feeding during neonatal period, (iv) motor stereotypies in the form of sideward movements of the head, (v) severe developmental delay, (vi) no speech, (vii) hyperactive and aggressive destructive behavior, and (viii) dysmorphic features such as flat mid face and depressed nasal bridge. However, the features are not distinguishing enough to raise the possibility of a specific syndrome.

### Truncation Mutation Causes Loss of Several Functional Element of LINS1 Protein

The truncating mutation identified in our study results in loss of a region of 368-757. The splice site mutation identified by Akawi et al. resulted in loss of 211-407 in LINS1. Thus, the region that is missing in both of these studies (368-407) could possibly a crucial region associated with cognition related functions (19). NextProt reports three phosphorylation sites at positions 591, 619, and 635, which were identified through mass spectrometry-based phosphoproteomic studies and predicted based on sequence similarities (NextProt accession: NX_Q8NG48). Of note, the truncation mutation also causes a loss of a ubiquitylation site at Lys407 (30). The loss of domains, intolerant sites/regions, evolutionarily conserved blocks of protein sequences, phosphorylation and ubiquitylation sites may all impact the function of LINS1 leading to the ID observed in the patients.

### Role of LINS1 in Development and Cognition

*LINS1* gene encodes for a human homolog of Drosophila segment polarity gene, lines (lin) which is known to be an essential regulator of the WNT signaling pathway and is involved in cognition (19). The human *LINS1* gene was first identified through bioinformatics methods by searching for WNT signaling molecules in Drosophila against Human EST databases and cloned to get a full length transcript (31). In addition to the brain, *LINS1* is expressed in human adult testis, spleen, prostate, skeletal muscles, thymus and in fetal kidney (31). Mechanistic studies in Drosophila revealed that *lin* is involved in regulating the Hedgehog and Notch pathway during embryogenesis (32). Consequently, *lin* is reported to play major role in the development of various organ such as epidermis (33), hindgut (34), foregut (34), muscles (35), testis-gonads (36), and brain-imaginal disk (37). *lin* is localized in the cytoplasm upon Hedgehog signaling and transported to the nucleus upon Wg signaling and may play role in transcription regulation by binding to DNA directly or to DNA bound protein complexes despite the fact that it does not have any DNA binding domains or nuclear localization signals (34, 38). It has been known to be a tissue and stage specific modulator of wingless signaling and essential for patterning of dorsal epidermis (38). WNT signaling is an evolutionarily conserved and ancestral pathway plays a critical role in embryonic development, organ function and maintenance (39). WNT signaling pathway act as a key regulator for central nervous system development and supports essential processes including neuronal polarization, neuronal migration and synaptogenesis (40, 41). Mutations identified in some of these molecules have been reported to disturb WNT signaling and reported to cause ARID (42, 43).

The major limitation of this study is the sample size. Owing to the rare nature of this disorder, we could not find additional families with *LINS1* mutations and patients having similar phenotypes. However, the genotype-phenotype correlation could be established based on the previously reported patients. More patients with similar phenotypes with *LINS1* mutations will support the association of *LINS1* with MRT27. Animal model studies proving effect of this mutation causing ID are warranted to definitely establish the causality of this disorder. Although, genotype/phenotype correlation could be established for the *LINS1* gene mutation using previously published studies, the premature birth of one patient with assisted vaginal breech delivery may have played some role, although it is still unlikely.

Another limitation of this study and indeed most studies from India is that there are no public repositories available for population SNP data representing different ethnicities in the Indian population. Indian population is still poorly represented in the publicly available population SNP frequency databases including 1,000 genomes project, ExAC, and gnomAD. Nevertheless, we have compared these databases to check the polymorphic nature of the mutations identified in our study and to identify the potential novel mutation that causes the disease. In order to check the polymorphic nature of the *LINS1* mutation, we checked whole exome sequencing data of >70 patients with ID, ataxia, Parkinson's disease and healthy unaffected relatives and we did not observe this mutation. These samples were collected from patients that predominantly represent the state of Karnataka. Our sequencing service provider which has a collection of SNPs obtained from 18,000 clinical exomes did not find this mutation. Thus, this variant is likely a novel variant not reported in the Indian population.

In conclusion, we report here the genetic cause for a rare autosomal recessive nonsyndromic ID, MRT27. In humans, the role of *LINS1* in brain development and synaptogenesis is still unexplored. Growing evidence in literature on autosomal recessive mutations in *LINS1* in patients with ID provide strong evidence for its involvement in brain development and cognition. Additional studies elucidating the function of *LINS1* will be required to understand the mechanisms by which *LINS1* causes ID. The detailed phenotypic characteristics and genotypes provided in this study should facilitate the diagnosis and management of this disorder.

## Data Availability Statement

The DNA sequencing data generated in this study is from patients and their relatives. We do not have consent from the concerned person to share the data in public repositories.

## Ethics Statement

The studies involving human participants were reviewed and approved by the National Institute for Mental Health and Neurosciences, Bangalore. We obtained informed written consent from the parents as the patients were minors and lack mental capacity to sign the documents. Written consent was obtained for the publication of this case report and any accompanying data (there are no images in our manuscript).

## Author Contributions

AP, SG, and BM designed the study, and revised and edited the manuscript. BM analyzed the data, interpreted the results, and wrote the manuscript. AB, AKB, and PP performed experiments. SG, KR, and AB performed clinical assessment and wrote parts of manuscript. BG wrote a section in the manuscript.

## Funding

This work was supported by the Wellcome Trust/DBT India Alliance Margdarshi Fellowship [grant number: IA/M/15/1/502023] awarded to Akhilesh Pandey. This work was funded under DBT-BioCARe scheme, Department of Biotechnology (DBT), Government of India (BT/PR18182/BIC/101/937/2016). BM is a recipient of DBT-BioCARe Women Scientist Award from DBT, Government of India. ArB is a recipient of Senior Research Fellowship from CSIR, Government of India.

## Conflict of Interest

The authors declare that the research was conducted in the absence of any commercial or financial relationships that could be construed as a potential conflict of interest.
